# Fluorescently Guided Optical Photothermal Infrared Microspectroscopy for Protein-Specific Bioimaging at Subcellular Level

**DOI:** 10.1021/acs.jmedchem.2c01359

**Published:** 2023-01-04

**Authors:** Craig Prater, Yeran Bai, Sabine C. Konings, Isak Martinsson, Vinay S. Swaminathan, Pontus Nordenfelt, Gunnar Gouras, Ferenc Borondics, Oxana Klementieva

**Affiliations:** † Photothermal Spectroscopy Corporation, Santa Barbara, California93101, United States; ‡ Neuroscience Research Institute, University of California, Santa Barbara, Santa Barbara, California93106, United States; § Medical Microspectroscopy, Department of Experimental Medical Science, Lund University, 22180Lund, Sweden; ∥ Experimental Dementia Research Group, Department of Experimental Medical Science, Lund University, 22180Lund, Sweden; ⊥ Division of Oncology, Department of Clinical Sciences, Wallenberg Centre for Molecular Medicine (WCMM), Lund University, 22180Lund, Sweden; # Division of Infection Medicine, Department of Clinical Sciences, Lund University, 22180Lund, Sweden; ∇ Synchrotron SOLEIL, L’Orme des Merisiers, 91192Gif Sur Yvette Cedex, France; ○ NanoLund, Lund University, 22180Lund, Sweden; ◆ Multipark, Lund University, 22180Lund, Sweden

## Abstract

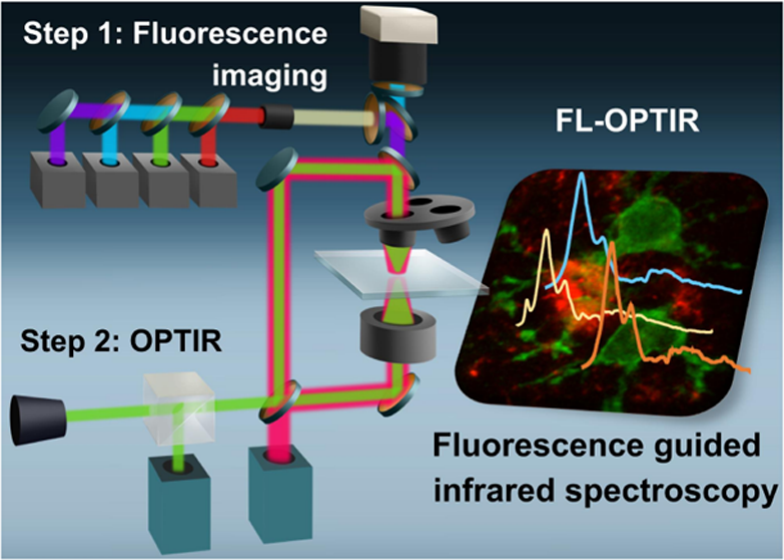

Infrared spectroscopic imaging is widely used for the visualization of biomolecule structures, and techniques such as optical photothermal infrared (OPTIR) microspectroscopy can achieve <500 nm spatial resolution. However, these approaches lack specificity for particular cell types and cell components and thus cannot be used as a stand-alone technique to assess their properties. Here, we have developed a novel tool, fluorescently guided optical photothermal infrared microspectroscopy, that simultaneously exploits epifluorescence imaging and OPTIR to perform fluorescently guided IR spectroscopic analysis. This novel approach exceeds the diffraction limit of infrared microscopy and allows structural analysis of specific proteins directly in tissue and single cells. Experiments described herein used epifluorescence to rapidly locate amyloid proteins in tissues or neuronal cultures, thus guiding OPTIR measurements to assess amyloid structures at the subcellular level. We believe that this new approach will be a valuable addition to infrared spectroscopy providing cellular specificity of measurements in complex systems for studies of structurally altered protein aggregates.

## Introduction

An increasing amount of evidence demonstrates that optical photothermal infrared (OPTIR) microspectroscopy is a valuable imaging and spectroscopy tool that can extract chemical information about biomolecules directly from individual cells and tissues at submicron resolution.^1−8^ OPTIR is a novel infrared spectromicroscopy technique that exploits the photothermal effect.^1^ Specifically, infrared (IR) absorption induces a photothermal response consisting of thermal expansion and a change in the index of refraction of the sample, which leads to an intensity change of the probe light and allows the detection of the photothermal response. The wavelength of the used probe beam is 532 nm, which is smaller than the wavelength of absorbed IR light (3–20 μm), thus providing a 5–10 times improvement of the spatial resolution.^9^ Similar to conventional infrared spectroscopy, OPTIR provides information about the molecular bonds and, thus, can be used to characterize the chemical composition of the sample to study protein structures. Both infrared microspectroscopy and OPTIR are nondestructive, structure-sensitive methods that have already been used in life science for in situ investigations of molecular structures^10^ and thus can be used for studies at the single-cell level, for example, to examine amyloid protein folding at subcellular locations.^11,12^

Structural changes of amyloid proteins that, under pathological conditions, may form β-sheet structures are considered the key pathological feature of many neuropathological diseases such as Alzheimer’s disease (AD);^13^ these β-sheet structures have a specific vibrational signature that can be detected by infrared spectroscopy.^14,15^ Several groups have experimentally demonstrated that OPTIR can be applied to live-cell imaging studies with exceptional chemical sensitivity.^1,10,16^ However, despite these promising results, the bottleneck of OPTIR is that when applied to a biological sample such as biological tissues or cells, it can be challenging to find specific areas of interest. This is because tissue sections and primary cell cultures can be composed of many cells of different types. Although it has been demonstrated that in vitro IR can discriminate between different cell lines,^17^ it is extremely challenging to perform IR analysis of biological tissues composed of multiple cell populations, for example, to analyze activated microglial cells in brain tissue. A method that could help to select the area of interest in a complex sample is fluorescent labeling of the target to guide OPTIR measurements. In this communication, we applied a hybrid technique combining epifluorescence imaging and OPTIR microscopy in a single instrument. Epifluorescence was used to rapidly locate regions for OPTIR measurements, which in turn provided rapid spectroscopic analysis of the chemical structure of fluorescently labeled regions of a sample, as well as neighboring unlabeled tissue. We refer to the new technique as “fluorescence-located” OPTIR or FL-OPTIR.

**Figure 1:**
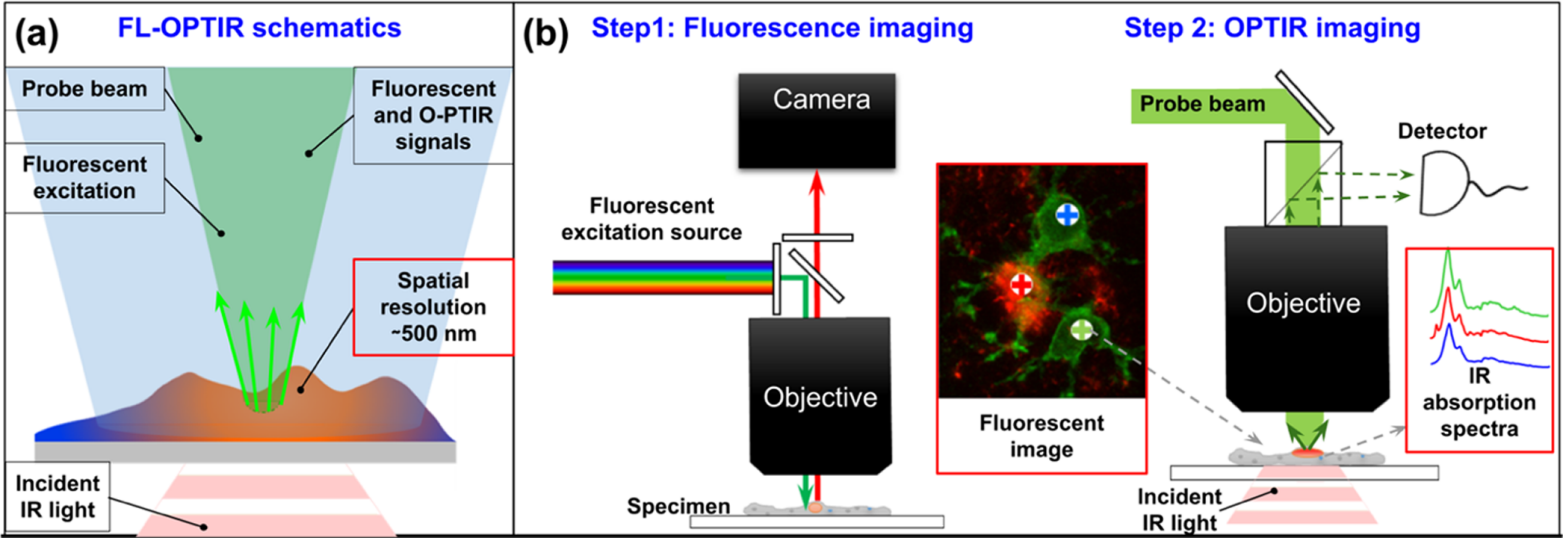
FL-OPTIR microscope configuration. (a) Schematic diagram of the working principle and lateral resolution for FL-OPTIR. The FL-OPTIR instrument employs 3 beams: (1) a 532 nm visible probe beam (green); (2) fluorescent excitation light (blue); and (3) a tunable IR pump beam (red). The photothermal response is detected as the modulation in collected probe light in response to the absorption of a pulsed IR beam. (b) Simplified schematic of the FL-OPTIR instrumentation. FL-OPTIR is performed as a two-step process. During the first step, the sample is excited by a fluorescence excitation source, and wide-field fluorescent emission images are collected by a camera. During the second step, the sample is illuminated by pulses of IR radiation at a series of different IR wavelengths while monitoring the modulation of collected probe light with a single-point detector.

For FL-OPTIR measurements, we used epifluorescence imaging to localize targeted labeled molecules, cells, or cellular organelles, followed by OPTIR to acquire spectra from identified molecules and cells. Although the use of fluorescent labeling to detect the protein of interest in tissue for infrared spectroscopy has been previously shown for synchrotron-based FTIR,^18^ the most important advantage of a single instrument setup is the match of spatial resolutions resulting in precise colocalization of two measurements. To illustrate the FL-OPTIR working principle, we provide a schematic diagram shown in Figure 1. In the FL-OPTIR instrument, we employed three beams: fluorescent light for epifluorescence imaging, a transmitted tunable IR pump beam, and a 532 nm visible probe beam to detect the IR absorption via photothermal detection. Specifically, if the IR pump beam is tuned to an absorption band of the IR illuminated region of the sample, IR absorption causes local heating that results in thermal expansion of the sample and an associated change in the index of refraction of the IR absorbing region of the sample. The thermal expansion and index changes cause a partial intensity change of scattered probe light, thus allowing to detect the photothermal response of the sample. The change in collected probe light is then used as an indicator of the amount of IR absorption by the sample, a key feature of the OPTIR approach, which provides improvement of spatial resolution based on the shorter wavelength of the probe beam. Using the Rayleigh criterion 0.61λ/NA for NA = 0.8, the resolution of the OPTIR technique is ∼400 nm, compared to 4.6 μm for a measurement at the amide I band using IR light alone. The spatial resolution of the OPTIR approach is also constant for all IR wavelengths, whereas traditional IR spectroscopy has a wavelength-dependent spatial resolution that worsens at long wavelengths/low wavenumbers. Although the elastic scattering is limited to the surface, the penetration depth of the OPTIR is defined by the reach of the IR beam (a few microns in the case of tissue^9^). Note that the FL-OPTIR implementation developed herein is distinct from prior studies by other groups that are based on IR detection of thermosensitive fluorophores,^19^ IR and two-photon excited fluorescence,^20^ and OPTIR used for temperature-dependent changes in fluorescence quantum efficiency.^21^ The FL-OPTIR approach employed here uses a single-element detector enabling high signal-to-noise spectroscopic measurements at each point on a sample, using fluorescent tags as a guide but not requiring fluorescence to produce an infrared absorption measurement.

## Results

**Figure 2:**
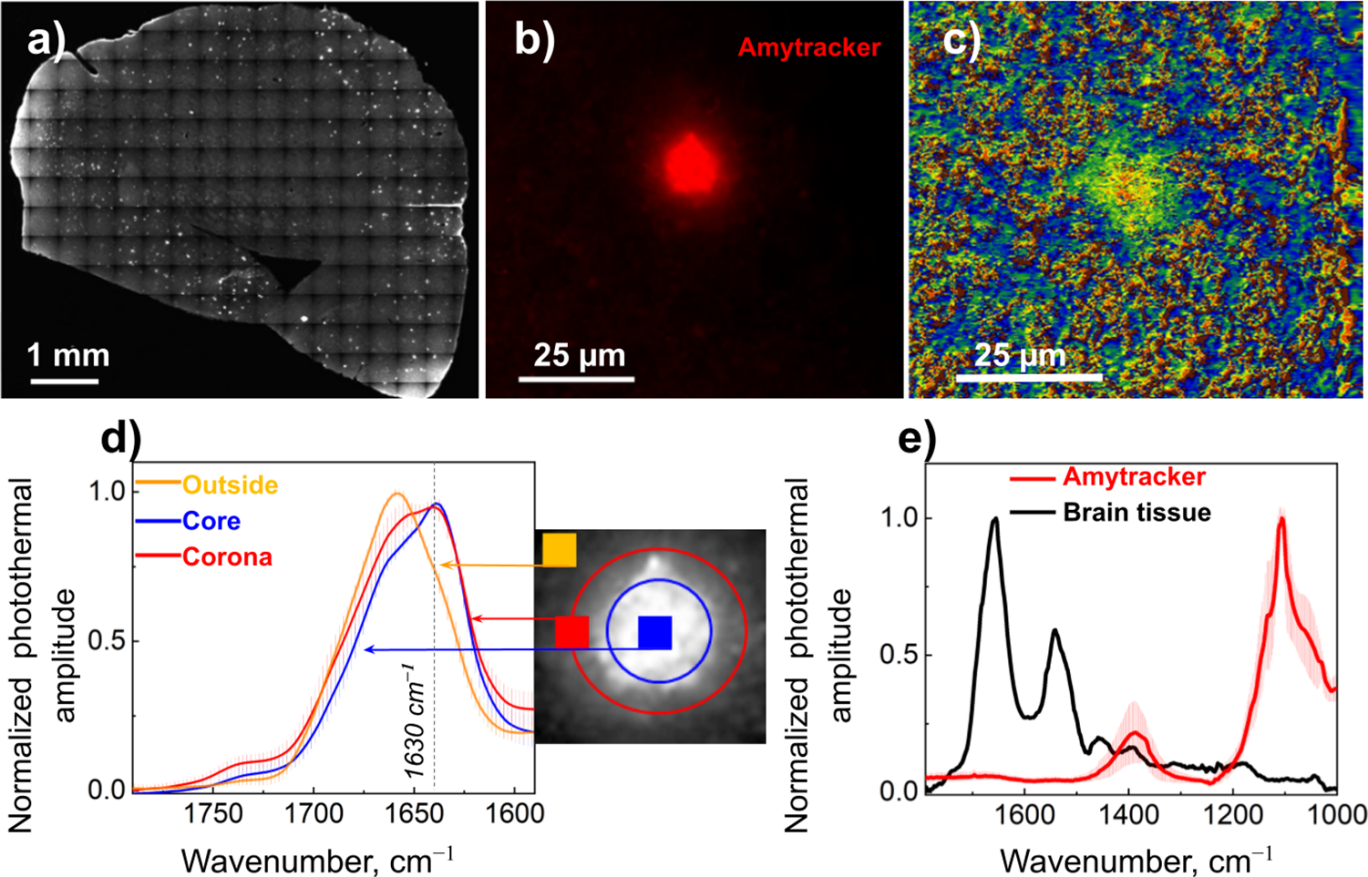
Imaging amyloid plaques in brain tissue by FL-OPTIR. (a) Fluorescent image of brain tissue with amyloid plaques labeled with Amytracker 520, overview. (b) Zoom of to the amyloid plaque stained with Amytracker. (c) Map demonstrating the distribution of β-sheet structures as a ratio of the bands at 1630–1656 cm^–1^ in the plaque shown in panel (b). (d) Averaged and normalized FL-OPTIR spectra were recorded from the outside, core, and plaque corona; the spectra locations were indicated by the markers of the corresponding color on the inset. Non-normalized, raw data are shown in Figure S4. (e) Comparison of representative IR spectra on brain tissue (black) and Amytracker fluorescent dye (red). Error bars in panels (d) and (e) represent standard deviation.

To demonstrate FL-OPTIR applicability for biospectroscopy, we provide three proof-of-concept experiments for structural analysis of β-sheet structures within an individual pathological structure in tissue (1); in specific cells in brain tissue (2); and in cultured primary neurons (3). We conducted measurements in two steps: first, wide-field epifluorescence imaging of specific regions of interest and then OPTIR measurements on the selected spots as well as around fluorescently labeled regions of the samples, thus demonstrating an approach that enables studies of molecular chemistry of specific molecules or the role of local environments on structural changes in individual cells in cultures and tissues. In Figure 2, we provide our first proof-of-concept experiment: OPTIR measurement of amyloid plaques in brain tissue. For this experiment, 12-month APP/PS1 transgenic mouse brains were chemically fixed and sliced into 16 μm sections on the microtome stained with Amytracker^R^ (Ebba Biotech, Solna, Sweden), a luminescent conjugated polyelectrolyte probe specific to amyloids.^22^ Tissue was mounted on a CaF_2_ window and air-dried (Figure 2a). An example of the bright-field image of the brain section of the same animal is provided in Figure S1, which shows that in brain tissue, amyloid plaques are mostly undetectable using bright-field observations. One of the strategies to detect amyloid plaques in label-free tissue can be a hyperspectral array of OPTIR spectra scanning over an extended area, as shown in Figure S2, and using the β-sheet associated IR absorption peak at 1630 cm^–1^ as a marker for amyloid.^14,15^ However, this blind strategy to locate plaques in brain tissues can be time-consuming, making it very challenging to study, for example, early AD stages when amyloid aggregates are sparsely distributed.^23^ Here, we show that Amytracker can be used to readily detect areas of interest and perform OPTIR measurements on the selected regions. Figure 2a,b shows fluorescently labeled amyloid plaque and distribution of β-sheet structures in the same plaque based on the ratio between two OPTIR maps acquired at 1630 and 1656 cm^–1^ (Figure 2c). Spectra acquired from selected locations based on fluorescence guidance are shown in Figure 2d, providing an example of amyloid plaque structural analysis. Specifically, the intensity corresponding to signals from α-structures and β-sheets can be compared within the plaque core and corona. Although amyloid plaques are believed to be formed by amyloids with high content of β-sheets, comparing fluorescence signal and elevations of β-sheet structure, it is possible to observe a high degree of heterogeneity in the distribution of β-sheet structures within the fluorescent spot. Thus, FL-OPTIR can be used to locate specific amyloid proteins and assess their structure in individual plaques directly in brain tissue. Figure S3 shows another type of analysis; here, a fluorescence image was used to select a specific area (line) for spectra acquisition, which was analyzed by the k-means clustering method. In our case, k-means clustering clearly showed the presence of two groups of spectra that have high and low amplitude at 1630 cm^–1^, and spectra with high 1630 cm^–1^ clearly colocalized with the fluorescent signal. Since the amyloid plaques in the tissue were stained with Amytracker, to test if this amyloid dye can affect spectra, we acquired OPTIR spectra from pure Amytracker deposited on CaF_2_ (Figure 2e). Spectral analysis showed that Amytracker does not have absorptions that may contribute to amide I and II regions, thus indicating that Amytracker can be used for fluorescent guidance of OPTIR measurements.

**Figure 3:**
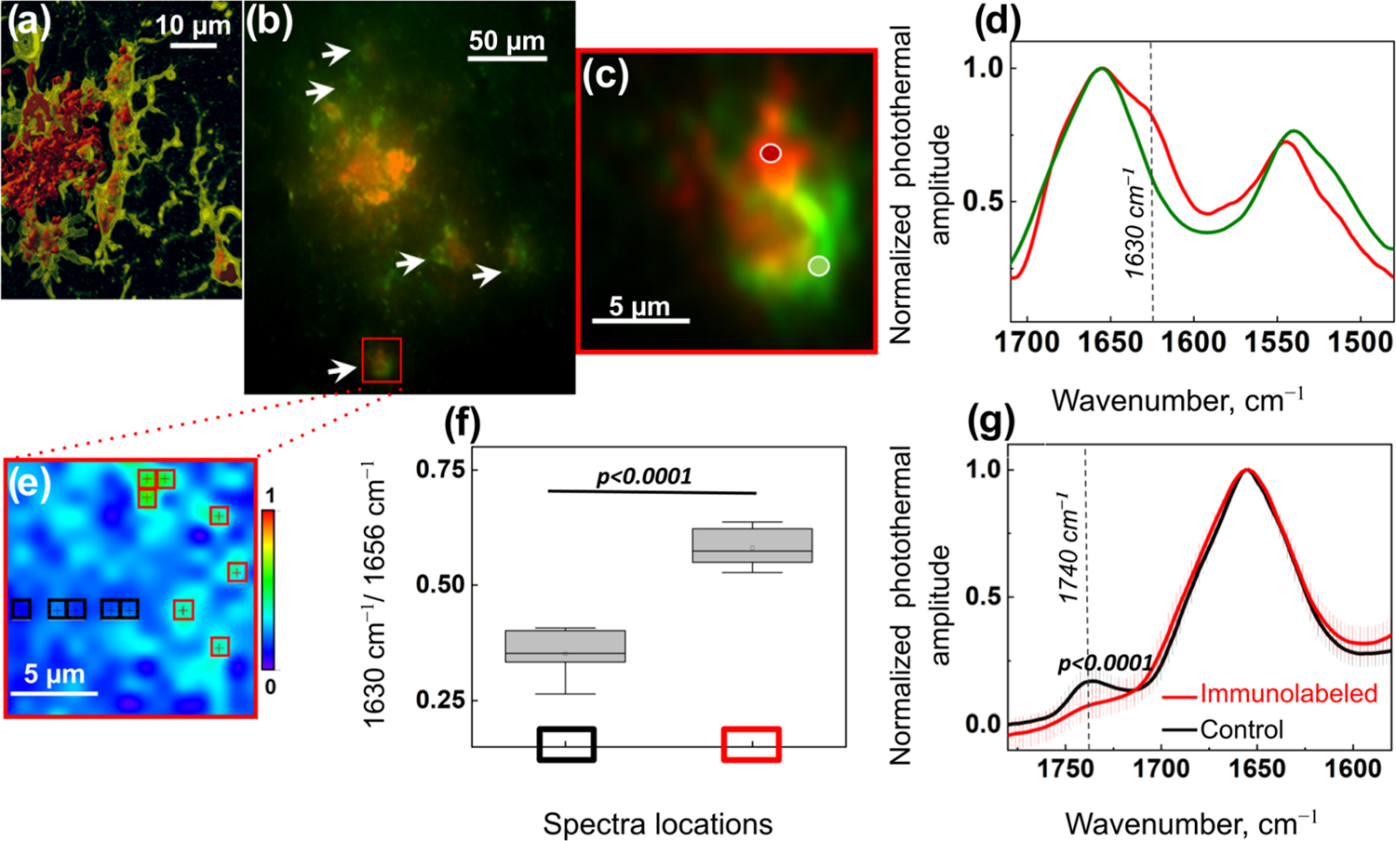
Imaging of microglia cells around amyloid plaques in brain tissue. (a) Example of microglial cell interaction with amyloid proteins. 3D confocal imaging was performed on 40 μm-thick brain tissue slices. Red iso-surfaces indicate immunolabeled Aβ, and semitransparent green surface shows microglial cells. (b) Example of FL-OPTIR fluorescent image of amyloid plaques in brain tissue showing amyloid proteins immunolabeled with Aβ specific antibody (82E1, red) and microglial cells immunolabeled with antibody Iba1 (green). Arrows indicate microglial cells. (c) Digital zoom of the microglial cell (red rectangle in (b)), dots indicate locations of spectra. (d) Averaged and normalized OPTIR spectra recorded from the locations indicated in panel (c), spectrum exhibiting β-sheets shown as red and control spectrum is green. The dashed line indicates a wavenumber for absorbance of β-sheets. (e) Slice of a hyperspectral map of IR absorption at 1630 cm^–1^ normalized to 1656^–1^ of the microglial cells shown in panel (c); square markers indicate the locations of spectra measured on regions with elevations of β-sheets (red) and Aβ-free regions (black). (f) Statistical analysis of OPTIR amplitude of the spectra locations, as indicated in panel (e), at 1630 cm^–1^. Tukey’s post-hoc test; *n* = 9; *n* represents the number of spectra. Data are represented as a mean ± s.d. (g) Averaged and normalized OPTIR spectra recorded from the tissue that was immunolabeled (red spectra) and tissue that was not immunolabeled (black spectra). Statistical analysis of the band centered at position 1740 cm^–1^ (−C=O, −COO absorption) Tukey’s post-hoc test; *n* = 20; *n* represents the number of spectra from different tissue spots. Data are represented as a mean, and error bars are ± s.d.

In the second experiment (Figure 3), we provide a methodological proof-of-concept experiment that demonstrates that FL-OPTIR can be used to study specific cell types in the tissue, which can be challenging for conventional IR microspectroscopy. For this purpose, we aimed to image microglia cells associated with amyloid plaques in brain tissue to assess their spectroscopic profile addressing the question of whether microglial cells can convert Aβ fibrils into monomers. For this experiment, we used 16 μm tissue sections labeled with Aβ-specific antibody 82E1, and microglial cells were immunolabeled with antibody Iba1 (Figures 3a,b and S5). Based on 3D analysis (Figure 3a), we estimated that 16 μm section thickness can fit the size of the cell body of individual microglia (16 μm tissue thickness is also optimal for both OPTIR and FTIR measurements). Using FL-OPTIR, it was possible to locate immunolabeled microglial cells in the vicinity of amyloid plaques and record OPTIR spectra. Figure 3c,d shows fluorescence image and corresponding OPTIR spectra indicating (1) the colocalization of microglia and amyloid-β, and (2) an elevation of the peak intensity at 1630 cm^–1^ indicative of β-sheet structures. Figure 3e shows the OPTIR hyperspectral map at the selected intensity of 1630 cm^–1^, and Figure 3f demonstrates elevations of β-sheet structures in microglia cells as compared to the adjacent area. To investigate if the primary antibody and secondary antibody used to detect amyloid-β may contribute to the spectra, we acquired OPTIR spectra from pure compounds that showed the peak centered at 1642 cm^–1^ (Figure S6a), which can possibly be assigned to unordered structures.^14^ Since no elevation of the intensity at 1630 cm^–1^ was recorded, we conclude that the IR signal from the antibody does not contribute to the signal of amyloid β-sheets (Figure S6b). However, it becomes evident that the band assigned to the ester group, 1740 cm^–1^, is decreased (Figure 3g). That phenomenon can be explained by tissue processing required for immunolabeling: the tissue was exposed to detergents for cell membrane permeabilization to facilitate the access for antibodies for immune reaction. Therefore, lipid ester groups were partially removed. Taken together, we show that 82E1 positive microglia cells exhibited elevated β-sheet content, indicating that microglia cells were associated with Aβ fibrils, thus demonstrating that FL-OPTIR is an indispensable approach when protein structural changes need to be addressed within the subpopulation of cells.

**Figure 4:**
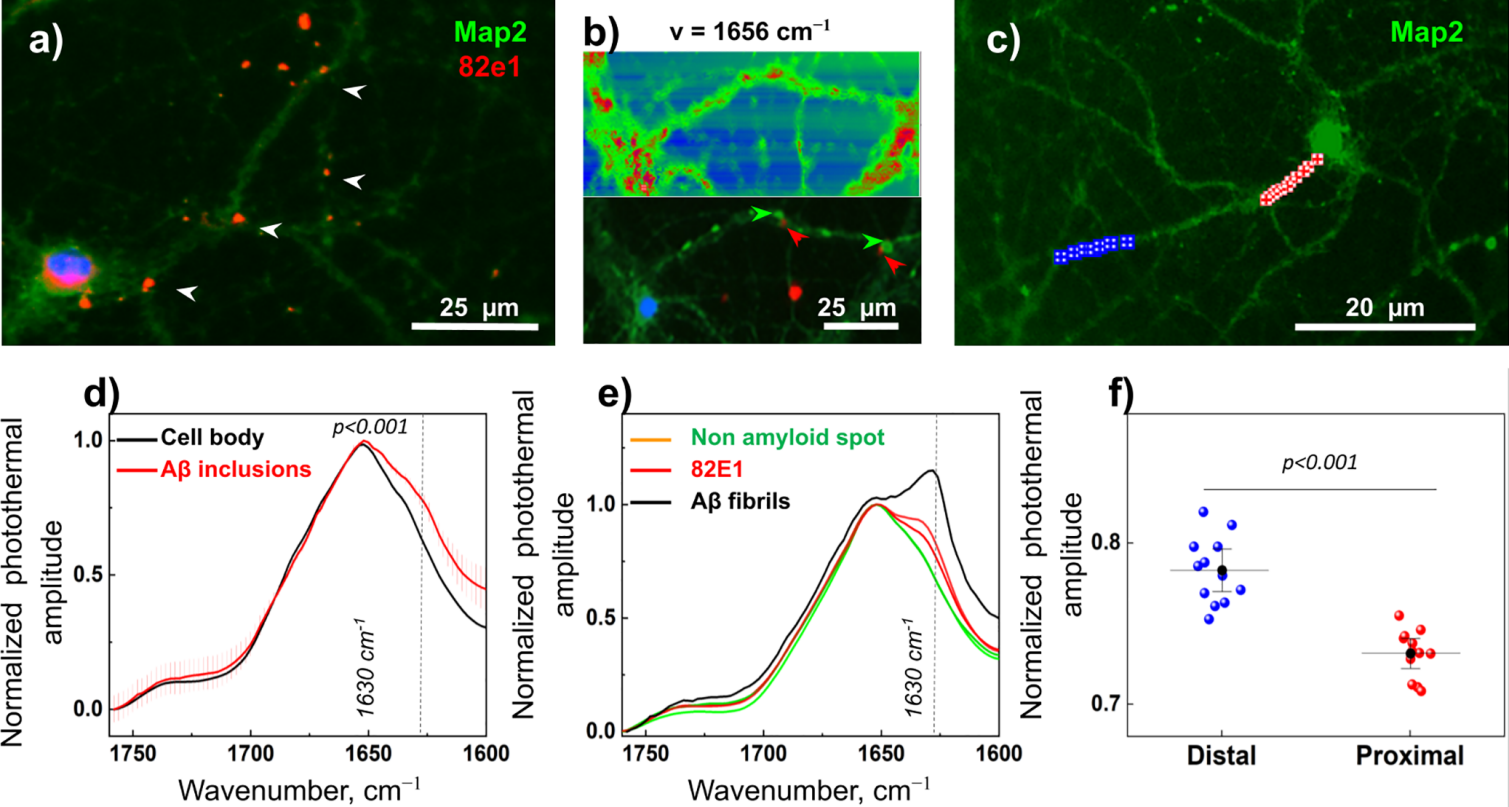
Structural imaging of Aβ (1–42) in primary neurons. (a) FL-OPTIR fluorescent image of primary neurons treated with Aβ (1–42). Cells were labeled with neuronal marker Map2, shown by green, while Aβ was labeled with the specific antibody 82E1 (red). (b) Single frequency map, which shows the distribution of the IR signal; below is shown a corresponding fluorescent image of amyloid proteins (red) associated with neurites (green); arrows indicate Aβ (red) and neurites (green). Corresponding scale image is shown in Figure S7c. (c) Another example of FL-OPTIR measurements: fluorescent image of primary neurons labeled with Map2 (green) treated with Aβ (1–42). Red and blue crosses show spectral positions that were acquired from proximal and distal parts of the neurite. (d) Averaged and normalized OPTIR spectra recorded from the cell body and red spots indicating added Aβ (arrows). The dashed line indicates a wavenumber for absorbance of β-sheets at 1630 cm^–1^; error bars show SD. (e) Right panel: averaged and normalized OPTIR spectra recorded from Aβ and associated neurites, as indicated by the arrow in the fluorescent image. The black spectrum represents Aβ (1–42) fibrils alone, used here as a positive control for β sheets. The dashed line indicates a wavenumber for absorbance of β-sheets at 1630 cm^–1^. (f) Normalized OPTIR amplitude from distal and proximal parts of neurite plotted at 1630 cm^–1^; Tukey’s post-hoc test; *n* = 10; *n* represents the number of spectra. Data are represented as a mean ± standard deviation.

In the third experiment (Figure 4), we investigated the applicability of FL-OPTIR for the structural imaging of Aβ (1–42) in cultured primary neurons. Similar to tissue studies, the structural heterogeneity of amyloid proteins makes it challenging to understand the relationship between neurotoxicity and the structure of Aβ; therefore, to assess amyloid structures directly in neurons, OPTIR is the technique that can provide structural information at subcellular resolution.^9,11,12,24^Figure 4 shows that FL-OPTIR can be used to guide spectroscopic measurements based on fluorescent signals and unambiguously discriminate between Aβ and other proteins (Figures 4a and S7). Moreover, we show that FL-OPTIR can be used to assess Aβ structures associated with neurites (Figure 4b). Figure 4c provides evidence that Aβ associated with the distal neurite part (after branching) contains more Aβ-aggregates compared to proximal. Further studies are needed to understand if Aβ is transported to distal neuritic parts or if these neuronal compartments are intrinsically more prone to bind Aβ.

## Discussion and Conclusions

Here, we introduce a new imaging method that combines fluorescent imaging and infrared spectroscopy to describe structural alterations within biological tissues. Through the development of a fluorescence-guided mid-infrared photothermal approach that allows targeting of specific cells, cellular organelles, and molecules in complex systems, we successfully address one of the most important obstacles in infrared spectroscopy of biological specimens. We provide proof-of-concept experiments that demonstrate the capability of FL-OPTIR to locate and analyze amyloid proteins and the associated microglia cells directly in tissue, which can address the role of the local environment in the progression of AD. To illustrate this, we performed imaging of microglia cells associated with amyloid plaques in brain tissue. We demonstrate that FL-OPTIR can be used to analyze specific cells and address the distribution of β-sheet structures at the subcellular level. Specifically, we show that using FL-OPTIR can help identify microglial cells associated with amyloid plaques and investigate fibrillar structures directly in microglia cells at the subcellular level. Finally, we investigated the applicability of FL-OPTIR for the imaging of cultured primary neurons treated with Aβ (1–42); neurons were grown directly on CaF_2_ spectroscopic windows, fixed immediately after the treatment, and air-dried. Similar to tissue studies, structural heterogeneity of amyloid proteins makes it challenging to understand the relationship between neurotoxicity and the structure of Aβ. By imaging amyloids and detecting their structures directly in neurons, we demonstrate significant methodological advances that allow for the detection and studies of vulnerable parts of neurons being attacked by amyloids. Thus, we believe that our new approach will be useful in the understanding of amyloid protein aggregation in complex systems such as tissues as well as in living cell studies. Since FL-OPTIR allows immediate identification of an area of interest, this significantly reduces the cell exposure time and thus could keep cells less stressed due to temperature changes or nutrient deprivation. Moreover, recent advances in OPTIR for imaging of living systems^10^ can be implemented for further FL-OPTIR studies to investigate the propagation of protein misfolding in living neurons, thus understanding how AD spreads from a diseased neuron to a healthy one. Thus, while immunofluorescence can be used to label biomolecules in tissues, it cannot perform in situ chemical structural analysis, which can be done by OPTIR, providing a spatial resolution of 500 nm. We developed and demonstrated a novel approach that simultaneously exploits OPTIR and epifluorescence to perform fluorescently guided IR spectroscopic analysis of proteins and cells in complex samples. By providing proof-of-concept experiments of structural analysis of β-sheet structures within an individual pathological structure in tissue (1); in specific cells in brain tissue (2); and in cultured primary neurons (3), we demonstrate the capability of FL-OPTIR to address important questions that are relevant for diseases caused by protein misfolding. We believe that this reported FL-OPTIR imaging technology promises broad applications, from monitoring amyloid structures to high-resolution mapping of cellular profiles directly in tissues, which goes beyond the reach of current infrared microscopy.

## Experimental Section

### General Information

Solvents and other reagents were purchased from either Thermo Fisher Scientific (Sweden) or Sigma-Aldrich (Sweden). Thus, all compounds are >95% pure and therefore were used without further purification.

All mouse experiments were compliant with the requirements of the Ethical Committee of Lund University.

### Preparation of Tissue

Medical microspectroscopy group: APP/PS1 mice (hAPPswe, PSEN1dE9)85Dbo/Mmjax (APP/PS1; Jackson Labs) were screened for the presence of the human APP695 transgene by PCR. Brain material, 16 μm sections, was collected using Leica Microtome, as described.^23^ Sections were labeled using antibodies and Amytracker manufacturer’s protocols (Ebba Biotech AB, Solna, Sweden). Before immunoreaction, neurons were permeabilized with 1% normal goat serum (NGS) (Thermo Fisher Scientific, Sweden), 1% bovine serum albumin, BSA, (Sigma-Aldrich, Sweden), and 0.1% TritonX (Sigma-Aldrich, Sweden) in phosphate-buffered saline (PBS) for 1 h. After membrane permeabilization, tissue sections were incubated with primary antibodies (overnight at 4 °C, using 1:1000 dilution in 1% BSA, 1% NGS in PBS). On the next day, tissue sections were washed three times for 15 min with PBS and incubated with secondary antibodies for 1 h at room temperature using 1:400 dilutions. After the reaction, tissue sections were washed with Milli-Q water, mounted on 0.2 mm CaF_2_, dried, and stored at 4 °C until measurements. FL-OPTIR measurements were at room temperature.

### Preparation of Primary Neurons

For culturing of primary neurons, we used APP knockout (APP-KO, Jackson Labs, Maine, U.S.A., JAX 004133) mice. These mice lack a functional APP gene; therefore, amyloid proteins, Aβ, and proteolytic cleavage products of APP are not expressed. Primary neurons were cultured following the ethical guidelines and were approved by the Lund University Ethical Committee (M46-16). Primary neurons were isolated from mouse embryos on embryonic day 16 using a well-established protocol described in Martinsson et al.^25^ Neurons were seeded directly on glass coverslips. We used a neurobasal medium with added glutamine, B27 (2%, Thermo Fischer Scientific, Sweden), and penicillin/streptomycin (Thermo Fisher Scientific, Sweden). Before seeding, coverslips were coated with poly-d-lysine of molecular weight >300 000 (Sigma-Aldrich, Sweden), followed by a wash in Milli-Q water. Neurons were seeded in 10% fetal bovine serum (FBS) and 1% penicillin–streptomycin in Dulbecco’s modified Eagle’s medium (DMEM; Thermo Fisher Scientific, Sweden) and incubated for 3–5 h for adhesion. After that, the media was exchanged for an FBS-free complete Neurobasal medium (#211103049, Gibco, Sweden). The complete neurobasal medium contains added 1.4 mM l-glutamine (#25030081, Gibco, Sweden), 1× B27 (#17504044, Gibco, Sweden), and 1% penicillin/streptomycin (SV300100, Thermo Fisher Scientific, Sweden). One embryo was used for one set of cultures; we used three embryos for the experiment. Neurons were treated with synthetic 1 μM Aβ (1–42) (Tocris) for 1 h and fixed with 4% paraformaldehyde (Sigma-Aldrich, Sweden) in phosphate buffer saline for 15 min at room temperature and washed three times with PBS. Before immunoreaction, neurons were permeabilized with 1% normal goat serum, 1% bovine serum albumin (Sigma-Aldrich, Sweden), and 0.1% Saponin (Sigma-Aldrich, Sweden) for 1 h. After membrane permeabilization, neurons were incubated with primary antibodies (overnight at 4 °C, using 1:1000 dilution). On the next day, neurons were washed three times for 15 min with PBS and incubated with secondary antibodies for 1 h at room temperature using 1:400 dilution. After the reaction, neurons were washed with Milli-Q water, dried, and stored at 4 °C until measurements. FL-OPTIR measurements were at room temperature.

### Optical Photothermal Infrared

OPTIR imaging was performed at Photothermal Corp, Santa Barbara. The IR source was a pulsed, tunable four-stage QCL device, scanning from 1800 to 800 cm^–1^ at 100 kHz repetition rate. The probe was a CW 532 nm visible variable power laser. The photothermal effect was detected through the modulation of the green laser intensity induced by the pulsed IR laser. Further details about the fundamentals of the technique and the instrument itself can be found in refs (17) and (19). Sample measurements were performed on dried samples in an instrument enclosure purged with dry nitrogen to minimize interference from IR absorption by atmospheric water vapor. Spectra were averaged for 5–20 scans. The collection parameters were: spectral range 1790–1400 cm^–1^, reflection mode at 2 cm^–1^ spectral resolution, to avoid photodamage IR power was set to around 1 mW maximum power at the sample (24% attenuator setting), the probe power was set to around 1.5 mW at the sample (3.4% attenuator setting). Background spectra were collected on a CaF_2_ reference sample with a low emissivity (“low-E”) coating using coating specifications described by DeVetter.^26^ OPTIR spectra were normalized to max at 1656 cm^–1^; second-order derivation of the spectra was used to increase the number of discriminative features; the Savitsky–Golay algorithm with a 5-point filter with third polynomial order was employed in this process. IR absorption images were obtained with a linear scan speed of around 100 μm/s with total acquisition times in the range of 5–30 min, depending on size and pixel resolution. The relative fraction of β-sheet structures was visualized by calculating the map intensity ratio between 1630 cm^–1^, a peak corresponding to β-sheet structures,^14,15^ and 1656 cm^–1^, maximum of amide I.^14^ The increase of intensity in the resultant ratio map was considered a sign of the higher concentration of amyloid fibrils. Fluorescence excitation was turned off for all OPTIR measurements, and fluorescence images were generally acquired first to avoid potential bleaching by the OPTIR probe laser.

### Fluorescence Visualization

To visualize Amytracker and signal from secondary antibodies, we used Alexa 488, MCHERRY, and DAPI filters installed in the OPTIR microscope (Photothermal Corp. Santa Barabara).

## Supporting Information Available

Examples of brain tissue used for the study; OPTIR measurements of amyloid plaques in brain tissue; structural analysis of individual plaque; and example of OPTIR spectra recorded from brain tissue and primary and secondary antibodies (Figures S1–S7) (PDF)

## Abbreviations

82E1: antiAβ antibody
FL-OPTIR: fluorescence-located optical photothermal infrared spectroscopy
Iba1: microglial marker
IR: infrared
Map2: neuronal marker
OPTIR: optical photothermal infrared spectroscopy

## Notes

All authors report that the funders had no role in the collection, analyses, or interpretation of data, in the writing of the manuscript, or in the decision to publish the results. All compounds, including buffers, were provided by manufacturers with stated purity of >95%.
