# Supplementary material for: Fluorescently Guided Optical Photothermal Infrared Microspectroscopy for Protein-Specific Bioimaging at Subcellular Level
Source: J Med Chem. 2023 Jan 4;66(4):2542–9. doi: 10.1021/acs.jmedchem.2c01359 (PMC9969395; doi:10.1021/acs.jmedchem.2c01359)
Supplement: Supplementary file 1 — jm2c01359_si_001.pdf [file jm2c01359_si_001.pdf]

Fluorescently guided optical photothermal infrared microspectroscopy for protein-specific bioimaging at subcellular level

Craig Prater,[1] Yeran Bai,[1,2] Sabine C. Konings,[3,8,9] Isak Martinsson,[4,9] Vinay S. Swaminathan,[5,8] Pontus Nordenfelt,[6,8] Gunnar Gouras,[4,9] Ferenc Borondics,[7] Oxana Klementieva[3,8,9*]

[1] Photothermal Spectroscopy Corporation, Santa Barbara, CA 93101, USA.

[2] Neuroscience Research Institute, University of California, Santa Barbara, CA 93106, USA.

[3] Medical Microspectroscopy, Department of Experimental Medical Science, Lund University, 22180 Lund, Sweden.

[4] Experimental Dementia Research Group, Department of Experimental Medical Science, Lund University, 22180 Lund, Sweden.

[5] Division of Oncology, Department of Clinical Sciences, Wallenberg Centre for Molecular Medicine (WCMM), Lund University, 22180 Lund, Sweden.

[6] Division of Infection Medicine, Department of Clinical Sciences, Lund University, 22180 Lund, Sweden.

[7] Synchrotron SOLEIL, L'Orme des Merisiers, 91192 Gif Sur Yvette Cedex, France.

[8] NanoLund, Lund University, 22180 Lund, Sweden.

[9] Multipark, Lund University, 22180 Lund, Sweden.

[*] *Corresponding_Author: email: oxana.klementieva@med.lu.se*

Supplementary Information Content:

Figure S1. Example of brain tissue used for the study.
Figure S2. Example of OPTIR measurements of amyloid plaques in brain tissue
Figure S3. Structural analysis of individual plaque.
Figure S4. Example of OPTIR spectra recorded from brain tissue.
Figure S5. Imaging of microglia cells around amyloid plaques in brain tissue
Figure S6. OPTIR spectra of primary and secondary antibodies.
Figure S7. Imaging of primary neurons.

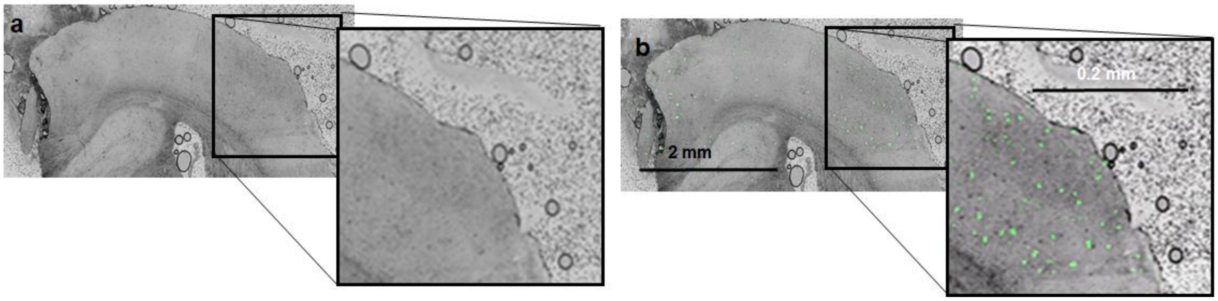

Figure S1. Example of brain tissue used for the study. a) Bright field overview of the brain tissue section, 12 months App/Ps1 mouse. **b**) Image of the section labeled with amyloid specific antibody 82E1 (green).

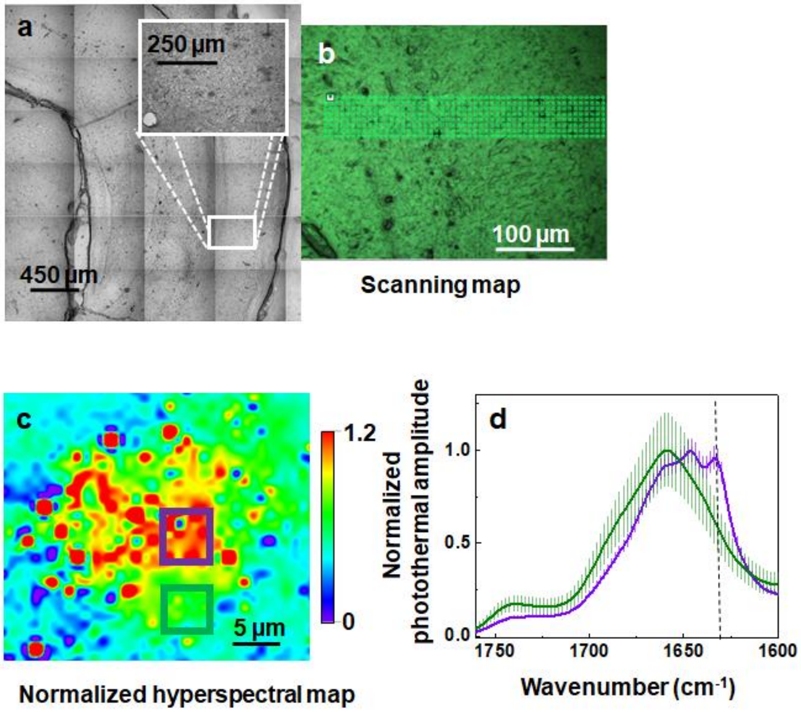

Figure S2. Example of OPTIR measurements of amyloid plaques in brain tissue. a) Bright-field overview of 16 µm brain tissue, inset shows optical densities that might be indicative of the presence of aggregated amyloids (though invisible in thicker tissue slides). **b**) Example of the mapping strategy to locate amyloid plaque using fast scanning, green crosses indicate spectral positions with step size 10 µm. **c**) Example of hyperspectral map of amyloid plaque at 1630 cm^{-1}; squares represent corresponding spectra locations shown in panel d. **d**) Averaged and normalized OPTIR spectra recorded from the locations indicated in c, spectra exhibiting β-sheets are shown as magenta (magenta square in c), and spectra from unaffected tissue are green (green square in c). Dashed line indicates a 1630 cm^{-1} wavenumber for absorbance of β-sheets. Error bars represent standard deviation.

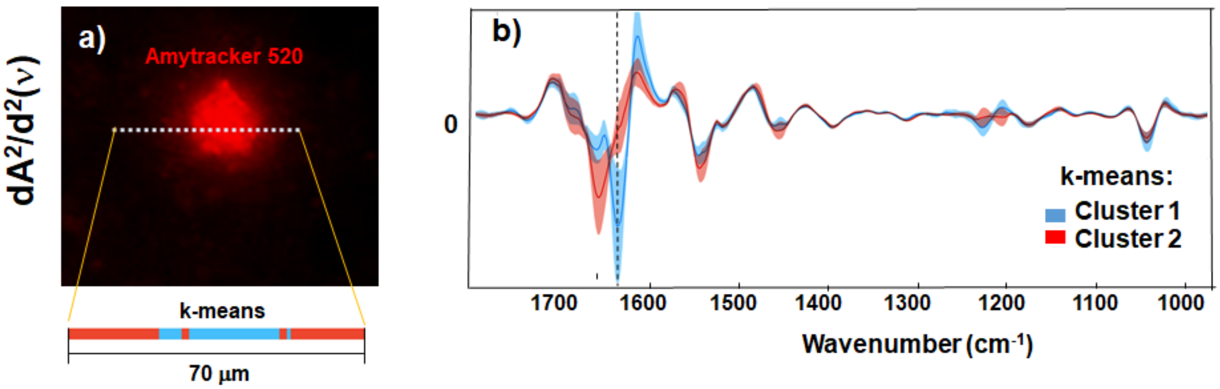

Figure S3. Structural analysis of individual plaque. a) Amyloid plaque stained with Amytracker 520 where the white line indicates a spectra location. The line below shows k-means (two clusters) based on spectra shown in (b). **b**) Second derivatives of OPTIR spectra acquired from a plaque shown in (a). Spectra exhibiting β-sheets are shown blue and spectra from unaffected tissue are red. Dashed line indicates a wavenumber for absorbance of β-sheets. Error bars represent standard deviation.

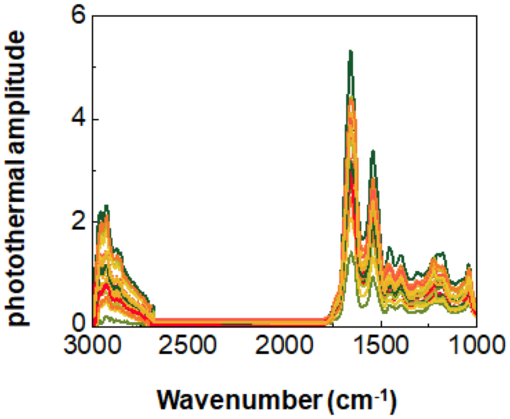

Figure S4. Example of OPTIR spectra recorded from brain tissue.

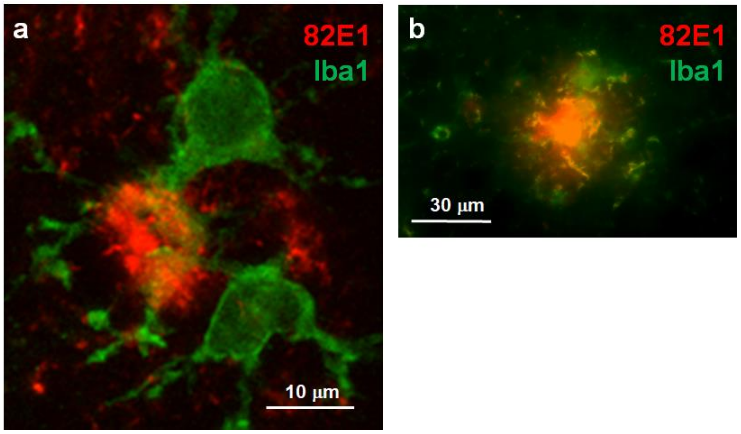

Figure S5. Imaging of microglia cells around amyloid plaques in brain tissue. a) Example of an epifluorescent image of amyloid plaques in brain tissue showing amyloid proteins immunolabeled with Aβ specific antibody (red) and microglial cells immunolabeled with antibody Iba1 (green). **b**) Amyloid plaques in APP/PS1 brain tissue used for FL-OPTIR.

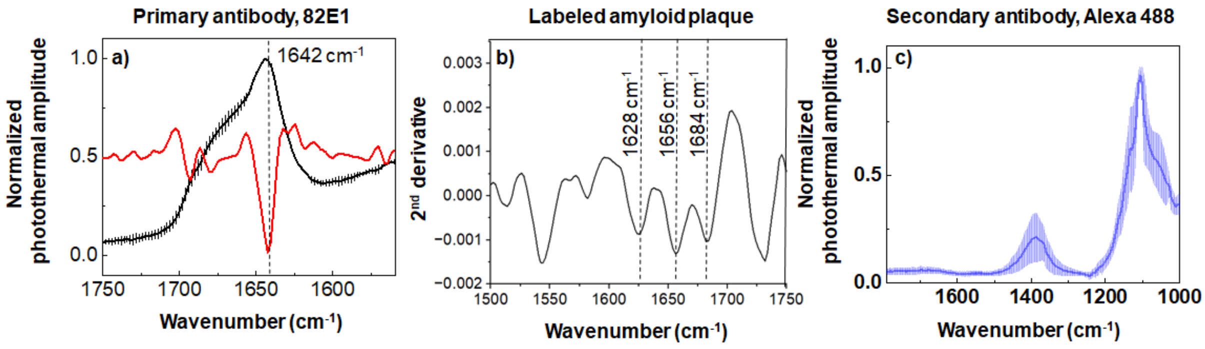

Figure S6. OPTIR spectra of primary and secondary antibodies. (**a**) Averaged and normalized OPTIR spectra recorded from primary antibodies. Redline in the left panel shows a second derivative used to calculate the exact peak position if the OPTIR spectra. (**b**) Second derivative of the spectra measured from amyloid plaque. (**c**) Averaged and normalized OPTIR spectra recorded from secondary antibody.

.

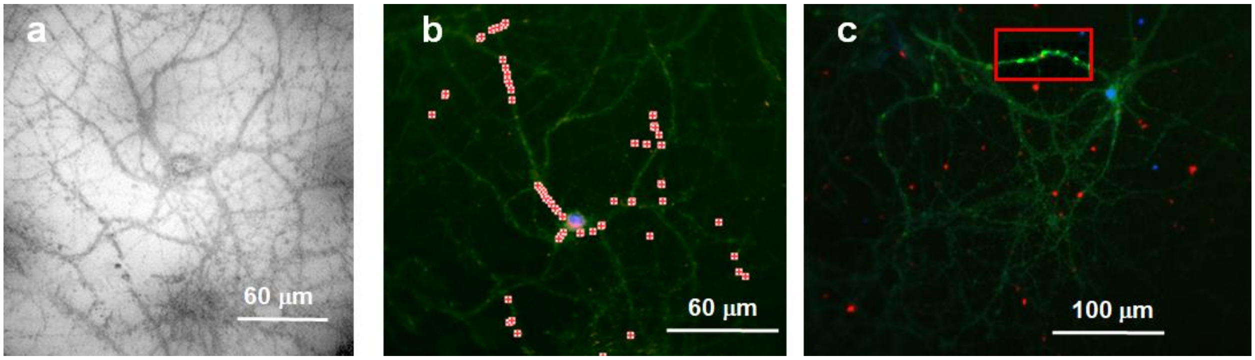

Figure S7. Imaging of primary neurons. a) A bright field image of neurons grown on a glass coverslip. **b**) Fluorescent image of primary neurons labeled with neuronal marker MAP2 shown by green and Aβ is shown by red. Arrows indicate spectral positions. c) Fluorescent image of primary neurons labeled with neuronal marker MAP2 shown by green and Aβ is shown by red. Red rectangle shows the map position for Figure 4 b.
